# Disseminated talaromycosis complicated by recurrent gastrointestinal bleeding and hemorrhagic shock: a case report

**DOI:** 10.1186/s12879-022-07230-8

**Published:** 2022-03-09

**Authors:** Xiaoya Cui, Feifei Su, Hui Ye, Yi Jiang, Xiuxiu Guo

**Affiliations:** 1 Department of Infectious Diseases, Wenzhou Center Hospital, Wenzhou, 325000 Zhejiang Province China; 2 Department of Pathology, Wenzhou Center Hospital, Wenzhou, 325000 Zhejiang Province China

**Keywords:** *Talaromyces marneffei*, Human immunodeficiency virus, Gastrointestinal hemorrhage, Hemorrhagic shock

## Abstract

**Background:**

Gastrointestinal involvement is not uncommon in patients with disseminated talaromycosis, but successful management of massive gastrointestinal bleeding and hemorrhagic shock secondary to talaromycosis is rarely reported. Clinical management strategies for these patients have not been well documented.

**Case presentation:**

Here, we reported a case of disseminated talaromycosis with recurrent gastrointestinal bleeding and hemorrhagic shock who was successfully alleviated solely with medical treatment.

**Conclusions:**

Early diagnosis and treatment for *Talaromyces marneffei*, intravenous fluid resuscitation, hemostatic therapy and blood transfusion are all essential for talaromycosis complicated with gastrointestinal bleeding and hemorrhagic shock. It is also necessary to warn about the possibility of bleeding induced or aggravated by endoscopic biopsy trauma.

## Background

Talaromycosis, which is an invasive fungal infection, was caused by *talaromyces marneffei* (previously named *Penicillium marneffei*) in immunocompromised hosts. Polulation with advanced human immunodeficiency virus (HIV) disease, and who reside in or travel to regions of endemicity such as Southeast Asia, East Asia and South Asia is generally susceptible [1–3]. *Talaromyces marneffei* disseminates in a hematogenous manner or via the lymphatic system throughout the body, involving the skin, and the respiratory, digestive and reticuloendothelial systems [4]. Given the extent of the lymphoid tissue throughout the gastrointestinal system, theoretically, it should be a common site of infection. However, few cases were reported on talaromycosis involvement in the digestive tract, and massive gastrointestinal bleeding and hemorrhagic shock secondary to talaromycosis are especially rare. In addition, how to successfully manage these patients is not well defined. Herein, we report a case of gastrointestinal talaromycosis complicated by repeated gastrointestinal hemorrhage and hemorrhagic shock in an HIV-infected patient.

## Case presentation

A 36-year-old man was admitted to our hospital for a 10-day history of abdominal pain and diarrhea on December 28, 2020. He had a history of HIV infection and has been taking lamivudine, tenofovir and efavirenz for 5 years. He had a resolved syphilis infection and no history of hepatitis B or C infection. One month before admission, the antiretroviral regimen was changed to lamivudine, tenofovir and lopinavir/ritonavir because of virologic failure at the local hospital. However, the regimen was changed back to the original regimens due to general discomfort 12 days later. Before admission, he has been treated with ceftizoxime sodium and metronidazole for 4 days in another hospital's surgery department but without clinical improvement. He lived in Yueqing, Zhejiang Province, located in the southeast of China.

**Fig. 1:**
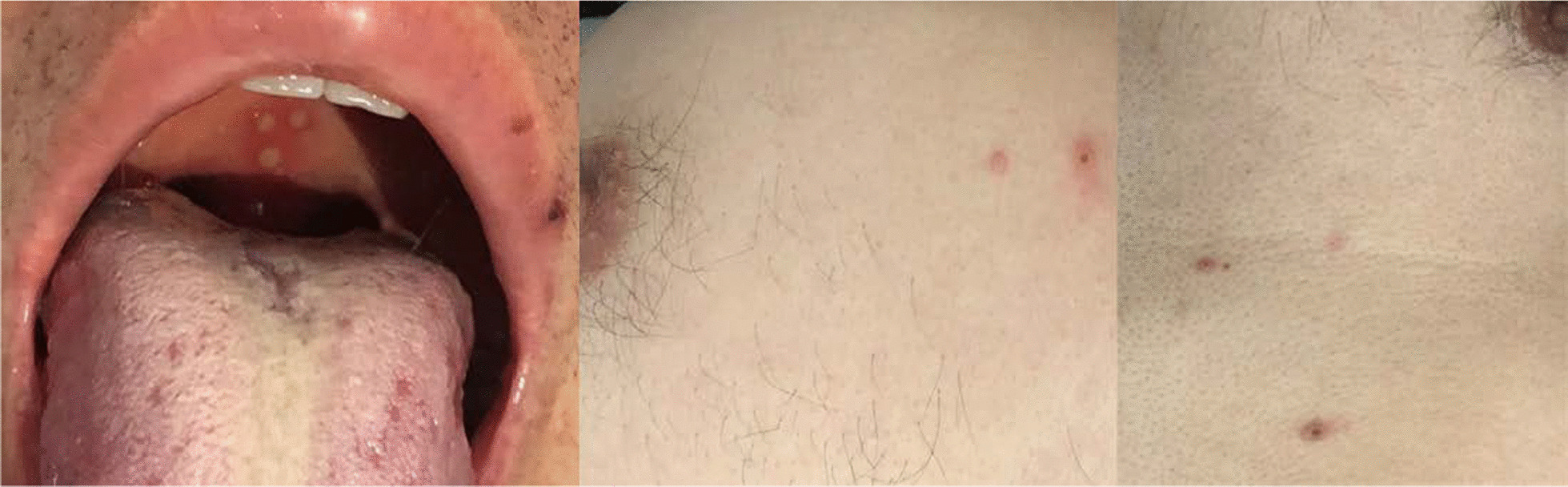
Skin lesions. There are approximately 10 small papules with central necrosis on his face and chest, three little painless ulcers on his palate

**Fig. 2:**
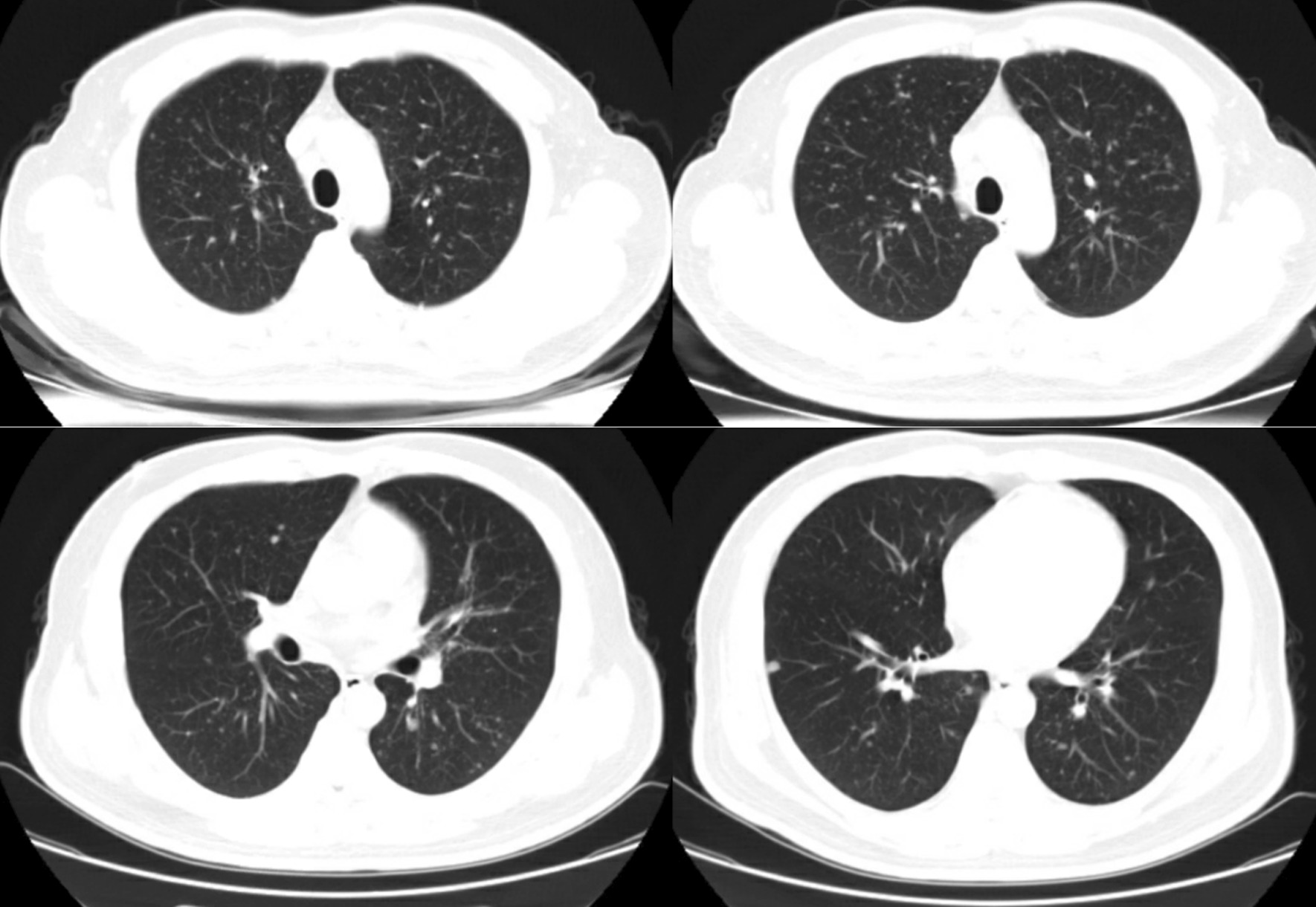
Presentation of chest CT scan. Chest scan showed diffuse small, well-defined nodules of different sizes in both lungs

Physical examination showed approximately 10 small papules with central necrosis on the face and chest, which were neither painful nor itching, and three little painless ulcers on the palate (Fig. 1). The remainder of the examination was unremarkable. Routine blood test results revealed leukocyte count at 3.9 × 10^9^/L, hemoglobin level at 117 g/L and platelet count at 95 × 10^9^/L. Abnormal biochemistry results were as follows: alanine aminotransferase, 132 U/L; aspartate aminotransferase, 133 U/L; albumin, 33.4 g/L; lactic dehydrogenase, 333 U/L; C-reactive protein (CRP), 45.3 mg/L. The fecal occult blood test was positive. Serum (1, 3) β-d-glucan measurement was normal, but serum galactomannan (GM) level was 4.07 µg/L. CD4 and CD8 T lymphocyte counts were 16 and 401 cells/mm^3^_,_ respectively. HIV RNA (viral load) was 1,270,000 copies/mL. Chest computed tomography (CT) showed small nodules disseminated throughout both lungs (Fig. 2). Abdominal CT showed extensive abdominal lymphadenopathy and some pelvic fluid. Brain CT was normal. Serum cryptococcal antigen was negative. *Toxoplasma gondii* IgG antibody was positive while IgM antibody was negative. Whole blood CMV-DNA and EBV-DNA were undetectable. Sputum acid-fast bacillus smear and T-SPOT.TB (a commercial interferon gamma release assay) were negative. Blood and sputum bacterial cultures were negative.

**Fig. 3:**
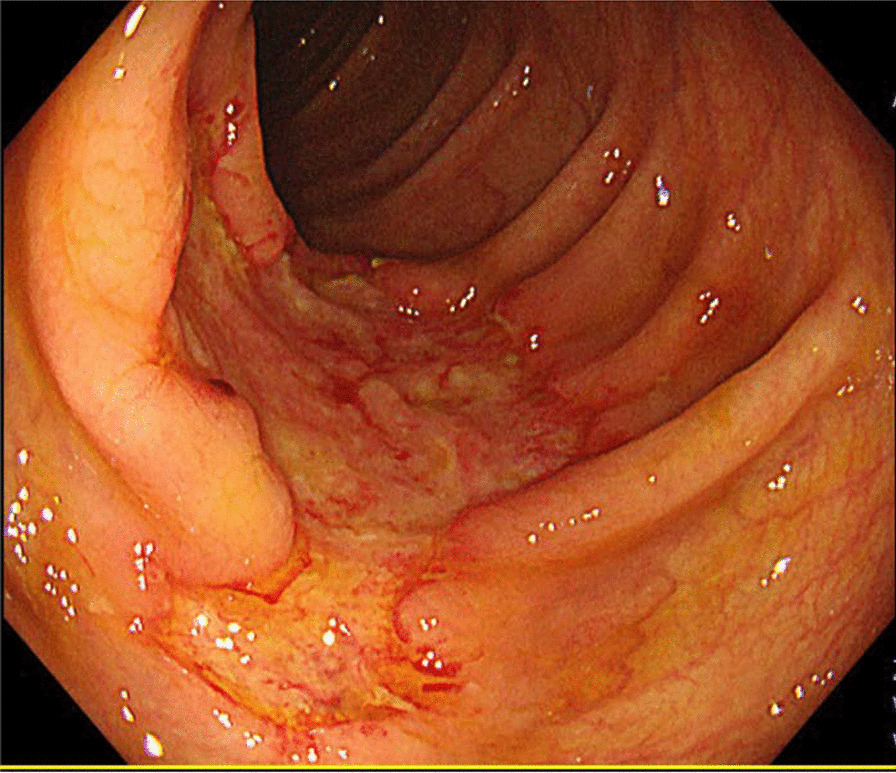
Presentation of colonoscopy. Colonoscopy showed an irregular marginal ulcer about 3 cm in size in the ascending colon

**Fig. 4:**
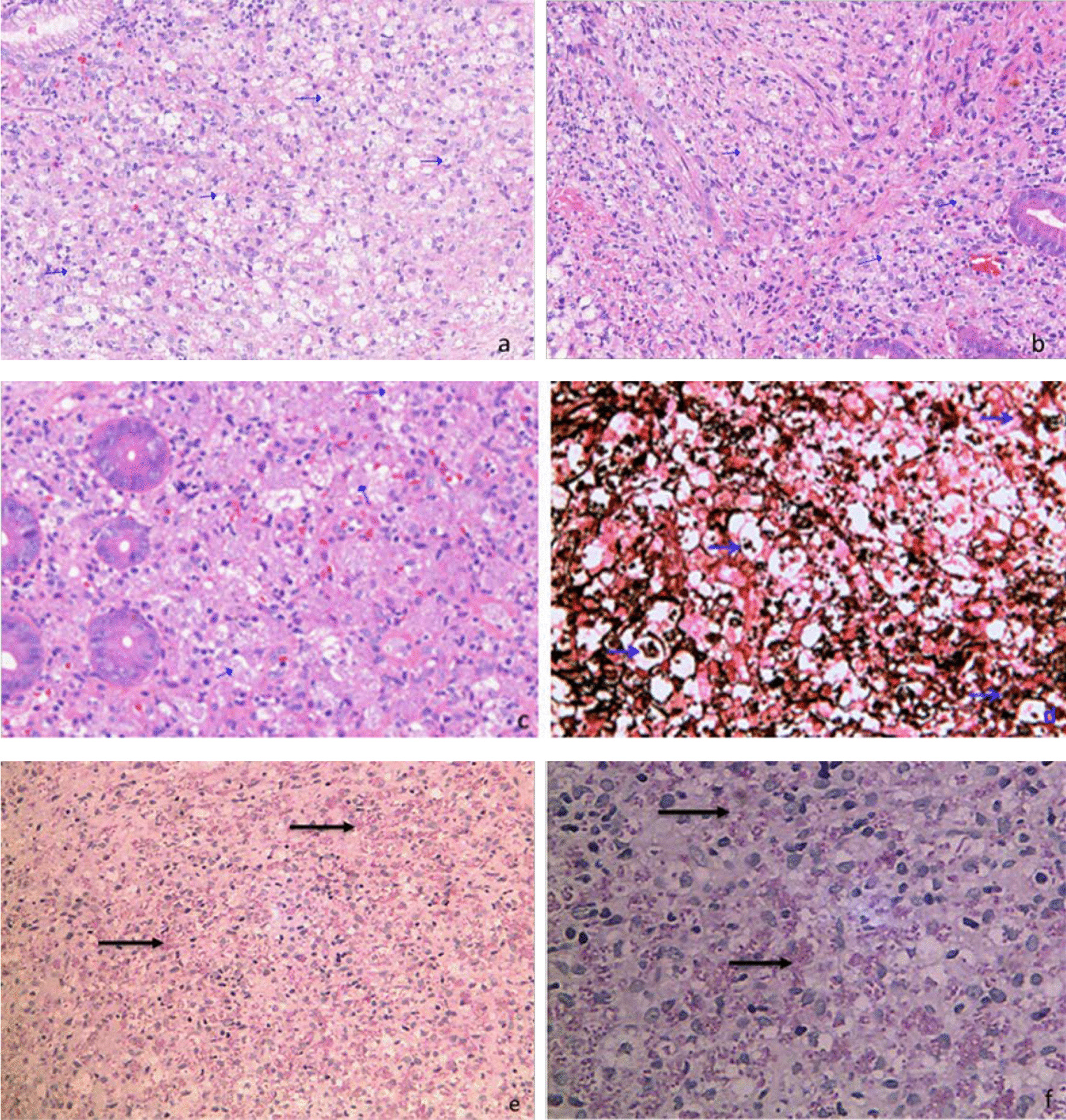
Histopathology of biopsy samples. Hematoxylin and eosin staining showing granulomas of macrophages were observed in the lamina propria of gastric body (**a**), ileocecum (**b**) and transverse colonic mucosa (**c**), and round or oval spores were observed in macrophages (× 200 magnification); Yeasts with positive Gomori’s methanamine silver nitrate staining (**d**) (400 × magnification) and Periodic acid–Schiff staining (**e**) (× 200 magnification), **f** (× 400 magnification) in macrophages

Talaromycosis was highly suspected, but no diagnosis-based antifungal therapy was given; only omeprazole was administered as a symptomatic treatment, and the abdominal pain was somewhat improved. Trimethoprim-sulfamethoxazole (0.96 g once a day) was provided for pneumocystis pneumonia and toxoplasmic encephalitis prophylaxis. After 7 days, gastrointestinal endoscopy showed inflammation of the descending duodenum and gastric mucosa, as well as multiple ulcers in the colon (Fig. 3). Two new papules with central necrosis on the neck were noticed. Then, diagnostic antifungal therapy with intravenous voriconazole (200 mg every 12 h) and antibacterial therapy with intravenous ceftriaxone sodium (2.0 g once a day) were administered. Antiviral therapy (lamivudine, tenofovir and efavirenz) was discontinued due to virological, immunological and clinical failures, as well as drug interactions between efavirenz and voriconazole. However, in the early morning of the next day, he presented gastrointestinal bleeding with about 500 mL bright red stool. Vital signs were monitored: breathing rate, 23 breaths per minute; heart rate, 106 beats per minute; blood pressure, 89/58 mmHg; normal temperature and oxygen saturation. Then, fasting, hemostasis (octreotide, esomeprazole sodium, hemocoagulase atrox) and intravenous fluid resuscitation were provided for symptomatic supportive treatment. Routine blood test results revealed leukocyte count at 2.8 × 10^9^/L, hemoglobin level at 69 g/L and platelet count at 43 × 10^9^/L. Suspended red blood cells, fresh frozen plasma and platelets were provided. Abnormal biochemistry results were as follows: alanine aminotransferase at 118 U/L, aspartate aminotransferase at 232 U/L, albumin at 28.7 g/L, CRP at 71.8 mg/L and procalcitonin (PCT) at 0.67 µg/L. The blood culture reported *Talaromyces marneffei* ten days after submission. Matrix-Assisted Laser Desorption/Ionization-Time of Flight (MALDI-TOF) was used for the identification of *Talaromyces* to the species level from cultured specimens. Histopathology of gastric, duodenal and colon biopsies indicated chronic active inflammation of the mucosa and a small amount of granulation tissue; in addition, fungal spores were observed in tissue cells, and the morphology was consistent with infection by *Talaromyces marneffei* (Fig. 4). Genotypic resistance results showed that the patient had nucleoside reverse transcriptase inhibitor resistance substitutions (A62V, K65R and M184I) and non-nucleoside reverse transcriptase inhibitor resistance substitutions (V106M, V179E and M230L). Then, dolutegravir plus lamivudine and zidovudine were administered, and intravenous ceftriaxone sodium was discontinued. However, in the morning of the next day, the patient had a sudden defecation of blood again, whose color was bright red and the recorded volume was about 500 ml. His blood pressure dropped to around 70/50 mmHg. Blood routine examination revealed leukocyte count at 2.8 × 10^9^/L, hemoglobin level at 57 g/L and platelet count at 58 × 10^9^/L. Prothrombin time was 16.4 s. Then, a multiple disciplinary discussion was held, with the following conclusion: suspension of antiretroviral therapy due to potential bone marrow suppression by zidovudine; changing voriconazole injection to amphotericin B deoxycholate antifungal therapy (since our hospital’s pharmacy did not have amphotericin B deoxycholate, we applied for a temporary purchase); strengthening symptomatic supportive treatment such as transfusion of red cells and plasma on the basis of hemostasis; considering the patient's condition, economic difficulties and surgical risk, endoscopic hemostasis, transcatheter mesenteric embolization and surgery are not recommended at that time. Over the next few days, blood test review (platelet count and liver function returned to normal) suggested talaromycosis was under control, but the patient continued to have persistent intestinal bleeding and hemorrhagic shock, and noradrenaline was administered to maintain blood pressure. On the morning of January 20, 2021, the patient requested to abandon treatment due to financial difficulties. After a brief discussion, he was provided free treatment, and amphotericin B deoxycholate 25 mg/day (0.5 mg/kg/day) was administered instead of voriconazole. Subsequently, his clinical symptoms gradually improved. Antiretroviral therapy with dolutegravir and lamivudine was initiated again and zidovudine was administered a few days later, with no obvious discomfort. After 4 weeks of antifungal treatment, CT of the chest and abdomen indicated that the lesions were significantly improved as well. Then, the patient was discharged from our hospital 39 days after admission, maintaining oral itraconazole 200 mg twice daily. He reported no recurrence of symptoms at 3 months follow-up, and itraconazole was reduced to 200 mg daily. A recent follow-up in June revealed that CD4 T lymphocyte cell count was risen to 215 cells/μL, and the antiretroviral regimen was changed to lamivudine/zidovudine and lopinavir/ritonavir due to financial difficulties, with no discomfort. He was very satisfied with the treatment outcome and expressed his profound gratitude.

## Discussion and conclusions

Disseminated infection involving multiple organ systems is the most common manifestation of talaromycosis in patients with advanced HIV disease. The infection frequently begins as a subacute illness characterized by fever, weight loss, hepatosplenomegaly, lymphadenopathy, and respiratory and gastrointestinal abnormalities [2, 5]. Gastrointestinal involvement presenting as diarrhea or abdominal pain occurs in 30% of patients [6]. However, few reports about talaromycosis involving the digestive tract have been published. Intestinal involvement in patients with disseminated talaromycosis might have been underdiagnosed because diagnostic yields concerning blood, bone marrow and skin cultures are sufficiently high to have rendered endoscopy-guided investigations of the intestine unnecessary [7].

The mortality of disseminated talaromycosis with antifungal therapy ranges from 10 to 30% [6]. In a few previously published papers, the majority of talaromycosis cases with gastrointestinal bleeding and hemorrhagic shock resulted in death [8–10], and experiences of successful treatment are not available. Bleeding in a case of gastrointestinal talaromycosis complicated by intestinal hemorrhage temporarily stopped after endoscopic hemostasis therapy with 1:1 epinephrine solution injected around the lesion. Intestinal bleeding had another two relapses and achieved spontaneous remission. However, hemorrhagic shock was not mentioned in this case [11].

This was a case of gastrointestinal talaromycosis complicated by repeated gastrointestinal hemorrhage and hemorrhagic shock successfully treated by internal medicine. His successful treatment could be attributed to the following factors. First, he was suspected of talaromycosis on the day of admission, and all tests revolved around confirming talaromycosis and screening for other diseases such as tuberculosis, cytomegalovirus disease, lymphoma, Crohn’s disease and so on. Secondly, in the absence of amphotericin, we administered voriconazole as a temporary, transitional antifungal treatment rather than waiting; as a result, related tests such as PCT, CRP, PLT and liver function were improved significantly before amphotericin B administration. Thirdly, when the patient had active gastrointestinal bleeding and hemorrhagic shock, intravenous fluid resuscitation and hemostatic therapy such as octreotide was started timely, and necessary blood transfusion was provided according to relevant guidelines [12–15]. Fourthly, when the patient declined the treatment, proper assessment of his condition prompted us to provide free treatment for a period of time.

However, there are still some points that deserve reflection and vigilance. First, the patient was highly suspected with talaromycosis on the day of admission but was not provided diagnostic antifungal therapy timely. The timing of diagnostic antifungal therapy needs to be further assessed and adapted according to the individual’s clinical situation. Secondly, in terms of examination, one could be more active in order to obtain early etiological diagnosis. Metagenomic next-generation sequencing may be a rapid and effective diagnosis approach [11, 16], although this test is quite expensive. Antigen detection such as Mp1p immunoassay and GM assay may also assist in the rapid diagnosis of *Talaromyces marneffei* infection [17, 18]. Thirdly, obvious gastrointestinal bleeding occurred in this patient only after gastrointestinal endoscopy, which indicates that the bleeding may be related to endoscopic biopsy trauma rather than the gastrointestinal talaromycosis itself. Whether it is necessary to perform prophylactic hemostatic therapy after gastrointestinal mucosal biopsy requires further study.

It is important to emphasize that when confronted with gastrointestinal hemorrhage and hemorrhagic shock, intravenous fluid resuscitation with crystalloids should be started on presentation. Blood transfusion shows both a decreased risk of rebleeding and a mortality benefit [12, 13]. Current guidelines recommend transfusion of red cells to maintain a hemoglobin level of more than 7 g per deciliter in most patients [14, 15, 19, 20]. If the patient's blood pressure is still low and life-threatening, appropriate intravenous infusion of noradrenaline, dopamine and other vasoactive drugs can be performed to temporarily maintain systolic blood pressure above 90 mmHg, to avoid prolonged blood perfusion insufficiency of vital organs and gain time for further rescue [20].

In conclusion, disseminated talaromycosis complicated by recurrent gastrointestinal bleeding and hemorrhagic shock is fatal. Early diagnosis and treatment of *Talaromyces marneffei*, intravenous fluid resuscitation, hemostatic therapy and blood transfusion are all essential for clinical management. Such cases often require multidisciplinary teamwork. It is also necessary to warn about the possibility of bleeding induced or aggravated by endoscopic biopsy trauma. Finally, any clinical decision must be taken according to the individual’s clinical situation and available resources.

## Data Availability

All data generated or analysed during this study are included in this published article.

## Abbreviations

HIV: Human immunodeficiency virus
CRP: C-reactive protein
GM: Galactomannan
PCT: Procalcitonin
CT: Computed tomography
